# Focusing light into scattering media with ultrasound-induced field perturbation

**DOI:** 10.1038/s41377-021-00605-7

**Published:** 2021-08-02

**Authors:** Zhongtao Cheng, Lihong V. Wang

**Affiliations:** Caltech Optical Imaging Laboratory, Andrew and Peggy Cherng Department of Medical Engineering, Department of Electrical Engineering, California Institute of Technology, Pasadena, CA 91125 USA

**Keywords:** Adaptive optics, Imaging and sensing, Biophotonics

## Abstract

Focusing light into scattering media, although challenging, is highly desirable in many realms. With the invention of time-reversed ultrasonically encoded (TRUE) optical focusing, acousto-optic modulation was demonstrated as a promising guidestar mechanism for achieving noninvasive and addressable optical focusing into scattering media. Here, we report a new ultrasound-assisted technique, ultrasound-induced field perturbation optical focusing, abbreviated as UFP. Unlike in conventional TRUE optical focusing, where only the weak frequency-shifted first-order diffracted photons due to acousto-optic modulation are useful, here UFP leverages the brighter zeroth-order photons diffracted by an ultrasonic guidestar as information carriers to guide optical focusing. We find that the zeroth-order diffracted photons, although not frequency-shifted, do have a field perturbation caused by the existence of the ultrasonic guidestar. By detecting and time-reversing the differential field of the frequency-unshifted photons when the ultrasound is alternately ON and OFF, we can focus light to the position where the field perturbation occurs inside the scattering medium. We demonstrate here that UFP optical focusing has superior performance to conventional TRUE optical focusing, which benefits from the more intense zeroth-order photons. We further show that UFP optical focusing can be easily and flexibly developed into double-shot realization or even single-shot realization, which is desirable for high-speed wavefront shaping. This new method upsets conventional thinking on the utility of an ultrasonic guidestar and broadens the horizon of light control in scattering media. We hope that it provides a more efficient and flexible mechanism for implementing ultrasound-guided wavefront shaping.

## Abstract (web summary)

A wavefront shaping technique with ultrasound-induced optical field perturbation is demonstrated, which provides an efficient and flexible mechanism to implement noninvasive and addressable optical focusing into scattering media.

## Graphical abstract

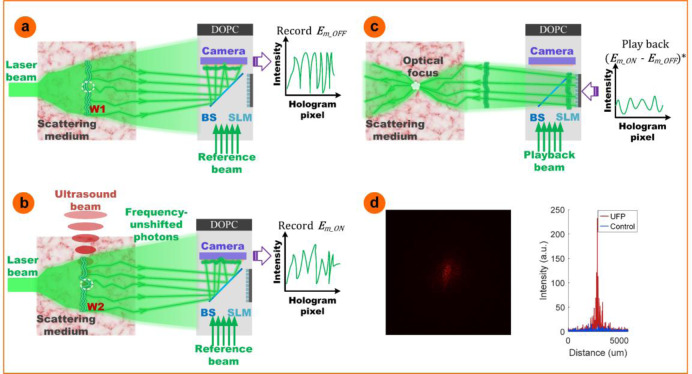

## Introduction

Focusing light efficiently into or through opaque scattering media is essential for many applications, including optical imaging, manipulation, therapy, and stimulation. However, optical scattering caused by microscopic refractive index inhomogeneities in scattering media randomizes the paths of the incident light, which creates a daunting challenge to the effective delivery of optical intensity. To overcome this challenge, wavefront shaping (WFS) methods are being actively developed and applied to focus light through or into scattering media. WFS counteracts optical diffusion by modulating the incident wavefront so that scattered photons experiencing different light paths constructively interfere at a target position. Depending on the technique used to determine the optimally modulated wavefront, WFS can be divided into three categories: feedback-based wavefront shaping^1–11^, transmission matrix inversion^12–17^, and optical phase conjugation (OPC)/optical time reversal^18–25^. The first two categories determine an optimally modulated wavefront through iterative processes that typically need thousands of measurements, which results in a long system runtime. OPC-based WFS methods directly measure the wavefront of a scattered field by interferometry and subsequently generate a conjugated version of the measured wavefront as the optimal incident wavefront. Therefore, OPC-based WFS methods can realize fast optical focusing into or through scattering media and show particular promise for applications involving dynamic samples.

Although focusing light through scattering media has attracted much current interest, focusing light into, rather than through, scattering media is both more useful and more challenging. For optical focusing into scattering media, a guidestar is generally required to provide feedback in finding the target wavefront^26^. Several types of guidestars have been employed, including fluorescence guidestars^27^, dynamic guidestars^28–33^, and ultrasonic guidestars^34–38^. Fluorescence guidestars and dynamic guidestars are invasive, and thus less desirable in general applications. Taking advantage of acousto-optic modulation to act as virtual light sources, ultrasonic guidestars show much promise for noninvasive optical focusing into scattering media.

The current technique for using an ultrasonic guidestar to perform optical focusing into scattering media is termed time-reversed ultrasonically encoded (TRUE) optical focusing, which was first proposed by our group in 2011^34^. Briefly, when scattered photons propagate through a focused ultrasound field inside a scattering medium, a fraction of the photons are frequency-shifted, and they are then called ultrasound-tagged photons. The optical field of the ultrasound-tagged photons is recorded and then time-reversed to generate an optical focus at the position of the ultrasonic focus (Supplementary Fig. S1). In fact, TRUE optical focusing shares the same essence as ultrasound-modulated optical tomography (UOT)^39–41^, an optical imaging technique assisted by ultrasound—both techniques detect and analyze frequency-shifted photons inside scattering media to define the acousto-optic interaction volume. It is well-known that the ultrasound-tagged photons, which are generally first-order ultrasound-diffracted photons, constitute only a small fraction of all the photons that pass through the focal area of the ultrasonic field^35,42–44^. Most photons remain frequency-unshifted and constitute the zeroth-order diffracted field, which is conventionally considered useless and even harmful in TRUE optical focusing. Given the fact of low optical tagging efficiency of ultrasound modulation, it is worth asking whether the frequency-shifted photons are the optimal choices to guide optical focusing. Might it instead be possible to guide the optical focusing using the more intense but “useless” zeroth-order diffracted photons? Although the conventional wisdom says no, here we report a new finding that challenges this thought.

In this work, we indeed leverage the zeroth-order diffracted photons from an ultrasonic guidestar as information carriers to guide optical focusing into scattering media. We find that the zeroth-order diffracted photons, although not frequency-shifted, have a field perturbation created by the existence of the ultrasonic guidestar. The perturbation originates mainly from the ultrasound-induced change of refractive index and scatterer displacement in the sample^42^. This perturbation can be detected by comparison with the same photons when the ultrasound field is absent. By detecting and time-reversing the differential field of the frequency-unshifted photons when the ultrasound is alternately ON and OFF, we can focus light to the position where the field perturbation occurs inside scattering media. We call this method UFP (ultrasound-induced field perturbation) optical focusing. The frequency-unshifted optical field, which is an impediment in TRUE optical focusing, now becomes an enabling factor in UFP optical focusing. This new method disrupts conventional thinking about the utility of an ultrasonic guidestar and broadens the horizon of light control into scattering media. Further, as we demonstrate experimentally, UFP optical focusing provides superior performance to conventional TRUE optical focusing, which benefits from the more intense zeroth-order photons. In addition, thanks to the differential detection scheme, we experimentally show that UFP optical focusing can be easily and flexibly developed into double-shot realization or even single-shot realization, which is significant for high-speed wavefront shaping.

## Results

**Fig. 1:**
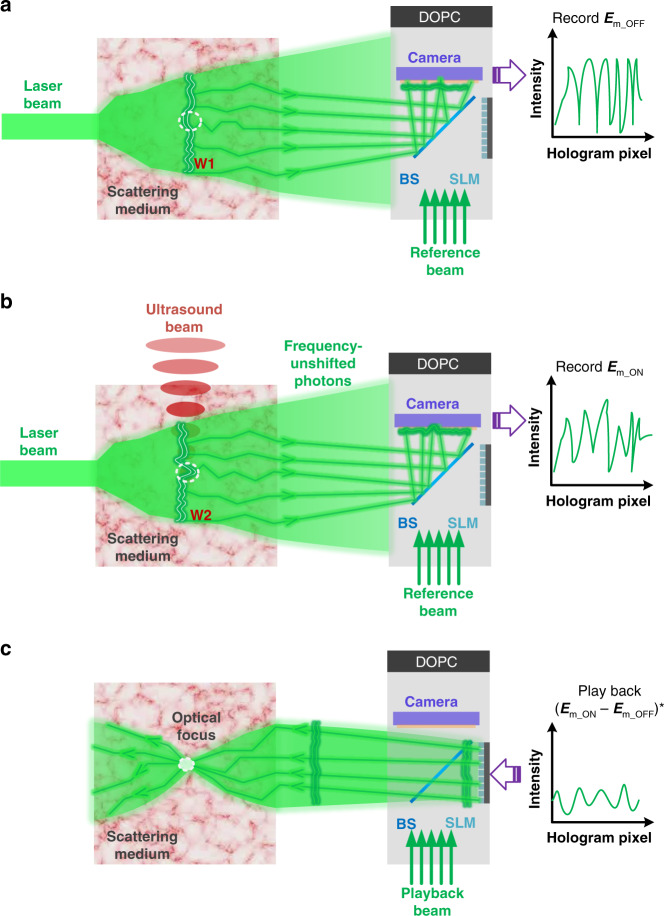
Principle of UFP optical focusing. In UFP optical focusing, we detect only the frequency-unshifted scattered photons because their higher energy dominates over that of the extremely weak frequency-shifted photons by an ultrasound field. **a** The scattered field through the scattering medium is directly measured via interference with a reference beam while the ultrasound is OFF. **b** Repeat the same measurement as in **a** while the ultrasound is ON. **c** The signals measured in **a** and **b** are different because of the ultrasound-induced field perturbation. Playing back the phase-conjugated differential field via a DOPC system generates a time-reversed beam that converges to the location where the perturbation occurs

The principle of UFP optical focusing is schematically illustrated in Fig. 1. When photons travel through an ultrasonic focus inside a scattering medium, they are diffracted into different orders. The frequency of the *n*th-order diffracted photons is *f*_0_ + *nf*_*a*_, where *f*_0_ and *f*_*a*_ are the frequencies of the incident photons and the ultrasound, respectively; *n* = 0, ±1, ±2,…. Generally, most of the energy of the diffracted light remains in the frequency-unshifted zeroth order, and the higher the diffraction order, the lower the diffraction energy. In other words, most photons reaching the detection plane are frequency-unshifted, which are contributed from two parts: one is from the zeroth-order diffracted photons by the ultrasound and the other one is from the photons that did not pass through the ultrasound volume. The first-order photons constitute a very small proportion of the total, typically 10^−4^–10^−3^ (see refs. ^35,43,44^).

Since the zeroth-order photons diffracted by the ultrasound have more energy than the first-order photons, it is natural to wonder whether we can leverage the zeroth-order photons to realize more efficient optical focusing into scattering media. This possibility has thus far been considered infeasible, because the zeroth-order photons have the same frequency as the photons that do not pass through the ultrasound focus, so they cannot be used to recognize the localized acousto-optic interaction volume. In this work, taking advantage of ultrasound-induced field perturbation and differential detection schemes, we make optical focusing based on the more intense zeroth-order photons possible. The operation steps of our UFP optical focusing are illustrated in Fig. 1a–c. First, the scattered field through a scattering medium, termed ***E***_*m_OFF*_, is directly measured in the camera plane of a digital OPC (DOPC) system by interference with a reference beam (Fig. 1a). Next, we launch a focused ultrasound pulse into the scattering medium and measure the scattered field again, now termed ***E***_*m_ON*_ (Fig. 1b). In this case, the measured field is contributed by the zeroth-order photons diffracted by the ultrasonic focus and the photons outside the ultrasonic focus. The two measured optical fields above are different because the ultrasound field induces an optical field perturbation around its focal position (see the areas denoted by white circles in Fig. 1a, b). The differential field, ${{{\boldsymbol{E}}}}_{m\_ON} - {{{\boldsymbol{E}}}}_{m\_OFF}$, cancels out the contributions from photons that do not pass through the ultrasonic focus, leaving only the perturbed field experienced by the zeroth-order photons. Finally, playing back the phase-conjugated version of the differential field generates a time-reversed beam that converges to the focal position of the ultrasound (Fig. 1c).

Mathematically, UFP optical focusing shares a similar framework as optical focusing based on dynamic guidestars, which is briefly described below. When the ultrasound is ON, the total optical field of the frequency-unshifted photons at the target plane can be denoted as ${{{\boldsymbol{E}}}}_t = {{{\boldsymbol{E}}}}_{t\_o} + {{{\boldsymbol{E}}}}_{t\_p}$, where ***E***_*t_o*_ is the original optical field when the ultrasonic field is absent and ***E***_*t_p*_ is the field perturbation induced by the ultrasound. Correspondingly, the measured optical field at the camera plane is ${{{\boldsymbol{E}}}}_{m\_ON} = {{{\mathbf{T}}}}{{{\boldsymbol{E}}}}_t$, where **T** is the transmission matrix describing the scattering medium whose elements follow a circular Gaussian distribution. Similarly, when the ultrasound is OFF, the measured field can be simply written as ${{{\boldsymbol{E}}}}_{m\_OFF} = {{{\mathbf{T}}}}{{{\boldsymbol{E}}}}_{t\_o}$. The differential field at the measurement plane is ${{{\boldsymbol{E}}}}_{m\_ON} - {{{\boldsymbol{E}}}}_{m\_OFF} = {{{\mathbf{T}}}}{{{\boldsymbol{E}}}}_{t\_p}$. Assuming time-reversal symmetry, playing back the conjugated differential field to the target plane yields ${{{\boldsymbol{E}}}}_{p}={{{\mathbf{T}}}}^{T}({{{\boldsymbol{E}}}}_{m\_ON} - {{{\boldsymbol{E}}}}_{m\_OFF})^{*}\, = {{{\mathbf{T}}}}^{T}{{{\mathbf{T}}}}^{*}{{{\boldsymbol{E}}}}^{*}_{t\_p}=\beta{{{\boldsymbol{E}}}}^{*}_{t\_p}$, where the superscript “*T* ” and “$\ast$” mean matrix transpose and conjugation, respectively. Here, we apply the approximated property of a transmission matrix of the scattering medium ${{{\mathbf{T}}}}^T{{{\mathbf{T}}}}^ \ast = \beta {{{\mathbf{I}}}}$, in which *β* is the fraction of the scattered light that is measured in the DOPC system and **I** is an identity matrix^26^. We see that the playback field ***E***_*p*_ is exactly the conjugated version of the perturbed field by the ultrasound, which can refocus at the location where the perturbation occurs. From the mathematical perspective above, we can also consider the ultrasonic guidestar in UFP optical focusing as a virtual dynamic guidestar, a counterpart of current physical dynamic guidestars. The physical dynamic guidestars, first proposed in time-reversed adapted-perturbation (TRAP) optical focusing^28^ and then developed to other forms such as magnetic particles^29^, microbubbles^30^, and photochromic proteins^31^, depend on implanted dynamic targets to create optical field variation inside scattering media. Also, because the location of a physical guidestar is not easy to be moved flexibly inside many scattering media, the time-reversed optical focus is not freely addressable, and two optical foci would appear if the original and the new locations of the guidestar are both inside the scattered optical field. Different from these physical dynamic guidestars, using ultrasound as a virtual dynamic guidestar here enables noninvasive and freely addressable optical focusing and does not have the double-focus disadvantage.

As shown schematically in Fig. 1, in UFP optical focusing, we detect only the frequency-unshifted scattered photons because their higher energy dominates over that of the extremely weak frequency-shifted photons. Thus, UFP optical focusing alleviates the challenge of extracting a tiny signal buried in a large background encountered in TRUE optical focusing. In addition, contrary to TRUE optical focusing, where the more intense zeroth-order diffracted photons are considered useless and even detrimental to the contrast of the time-reversed focus, in UFP optical focusing, the zeroth-order diffracted photons are not an impediment but instead the real information carriers, which renders a new and more efficient working mechanism of an ultrasonic guidestar.

**Fig. 2:**
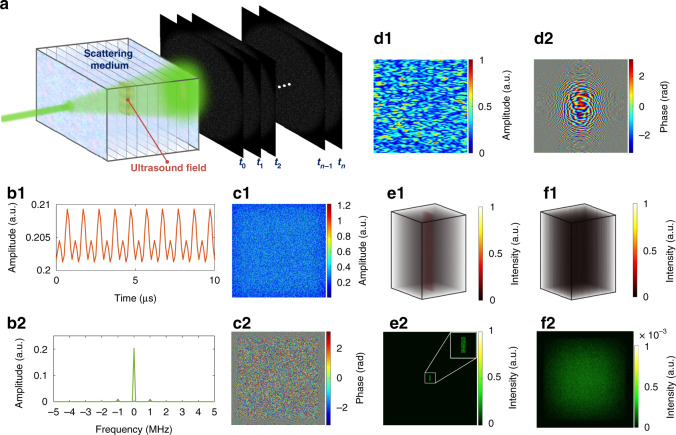
Conceptual simulations of UFP optical focusing into scattering media. **a** Schematic of the simulation model. **b1** Amplitude time sequence of the optical field at a given pixel of the observation plane when the ultrasound field is ON. **b2**, Fourier transform amplitude of the time sequence in **b1**. **c1**–**c2** Amplitude and phase maps, respectively, of the frequency-unshifted optical field in the observation plane when the ultrasound is ON. **d1**–**d2** Amplitude and phase maps, respectively, of the complex differential field between the frequency-unshifted photons when the ultrasound is on the ON and OFF states. The amplitude map is normalized here. **e1** A 3D representation of the time-reversed optical intensity in the scattering medium, using UFP optical focusing. The intensity is normalized. **e2** Time-reversed optical focus inside the scattering medium. Inset, the zoomed-in view of the focus. **f1** A 3D representation of the optical intensity in the scattering medium when playing back a random optical wavefront. The intensity is normalized to the maximum intensity of the time-reversed optical focus in **e1**. **f2** Only speckles can be seen at the focal position of the ultrasound when playing back a random optical wavefront

To gain a better understanding of the ultrasound-induced field perturbation for the frequency-unshifted photons as well as the concept of the UFP optical focusing, we developed a simplified model to simulate the working steps illustrated above. In the model, a scattering medium is simplified as many thin scattering layers uniformly separated by a tiny distance (Fig. 2a, detailed in “Materials and methods”). A Gaussian randomly distributed refractive index map is allocated to each scattering layer, with a pre-designated mean and standard deviation. In the middle of the scattering medium, an acoustic pressure distribution, described as $P\left( {{{{\boldsymbol{r}}}},t} \right) = P_0{{{\mathrm{sin}}}}(\omega _at - {{{\boldsymbol{k}}}}_{{{\boldsymbol{a}}}}{{{\boldsymbol{r}}}})$, is generated to approximate the ultrasonic field in the focal area of a focused ultrasound transducer. Here, *P*_0_ is the ultrasonic peak pressure, $\omega _a$ is the acoustic angular frequency, ***k***_*a*_ is the acoustic wave vector, and ***r*** is the position vector. The acoustic pressure induces refractive index changes in the scattering medium, ${\Delta} n = n_0\frac{{\partial n}}{{\partial p}}P\left( {{{{\boldsymbol{r}}}},t} \right)$, where $n_0$ is the original refractive index without the ultrasound field and $\frac{{\partial n}}{{\partial p}}$ is the adiabatic piezo-optical coefficient of the medium. Therefore, at each sampling time *t*, the refractive index at the ultrasonic volume becomes $n_0 + {\Delta} n$. A field-propagation method (see “Materials and methods”) is employed to calculate the optical fields in the observation plane at a series of sampling times $t_0$, $t_1$,…$t_n$. The time sequence of the optical fields at each pixel of the observation plane is Fourier transformed to obtain the amplitude and phase of different frequency components of the photons. Examples of such as a time sequence and the corresponding Fourier transform amplitude are shown in Fig. 2b1, b2, respectively. It is obvious that most photons keep their original frequencies despite the ultrasound modulation. As such, we extract the amplitude and phase of the frequency-unshifted photons at the examined pixel position. We repeat this calculation for all the pixels within the observation plane to obtain the complex field of the frequency-unshifted photons when the ultrasound is ON (Fig. 2c1, c2). Then the ultrasound field is removed from the scattering medium (i.e., the ultrasound is OFF), and the optical field in the observation plane is directly calculated by using the field-propagation method. Comparing the frequency-unshifted optical fields when the ultrasound is ON and OFF, we find that they are not the same. The differences between them are shown in Fig. 2d1, d2 for amplitude and phase, respectively, illustrating the ultrasound-induced field perturbation. Comparing Fig. 2d1 with Fig. 2c1, it can be seen that the differential field has much larger speckle sizes, which is expected because the differential field is contributed by a much smaller virtual light source, i.e., the relatively compact volume perturbed by the ultrasound. Finally, we back-propagate the wavefront of the conjugated differential field to all the planes within the scattering medium. A 3D representation of the time-reversed optical intensity in the scattering medium is shown in Fig. 2e1, and a close-up image of the optical intensity at the ultrasound position is shown in Fig. 2e2. The compact central focal spot in Fig. 2e2 demonstrates the effectiveness of the UFP optical focusing. As a control, we also played back a random optical wavefront. In this case, only optical speckles in the volume of the scattering medium can be seen, as shown in Fig. 2f1, f2. See the “Materials and methods” section for more details about the simulations.

It should be noted that our model considers only the refractive index modulation due to the presence of acoustic pressure. In fact, besides refractive index modulation, an ultrasound field can also cause particle displacements^42^ and shear waves^45^, which may be other sources of optical field perturbation inside the ultrasonic volume. These factors are too complicated to be considered simultaneously in the model. Even so, the simplified model provides a helpful way to verify and illustrate the proposed method computationally.

**Fig. 3:**
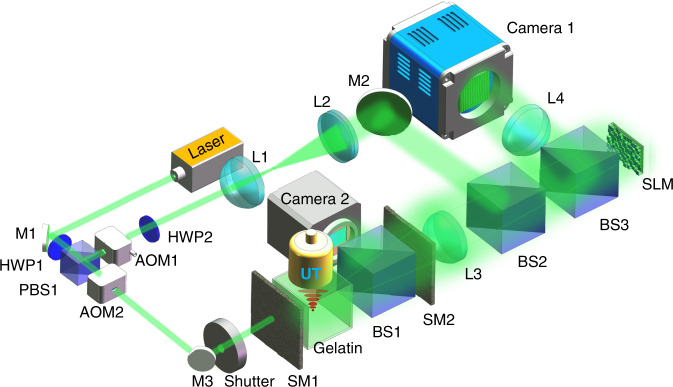
Schematic of the experimental setup. AOM acousto-optic modulator, BS beam splitter (nonpolarizing), HWP half-wave plate, L lens, M mirror, PBS polarizing beam splitter, SLM spatial light modulator, SM scattering medium, UT ultrasound transducer

To demonstrate the UFP optical focusing experimentally, we built a DOPC system that works as a wavefront recording and playback engine, as shown in Fig. 3 (see “Materials and methods” for more details). The DOPC system is a Mach–Zehnder interferometric configuration. A gelatin cube is sandwiched between two scattering media (SM1 and SM2) to act as the sample in experiments. The gelatin cube is submerged in a water tank to provide sufficient ultrasound coupling. In the recording step, the collected light scattered through the sample interferes with a plane reference beam at the SLM plane. The interference hologram is relayed to a camera (Camera 1) placed at the conjugated plane of the SLM for recording. Different operation modes of UFP optical focusing have different recording requirements, which will be described later. In the playback step, a phase map based on the corresponding data analysis method is loaded on the SLM to modulate the plane reference beam, which plays back to the sample. To validate that light is indeed focused into the sample, a beam splitter is inserted between the two scattering media to divert a copy of the playback beam to another camera (Camera 2) for verification.

**Fig. 4:**
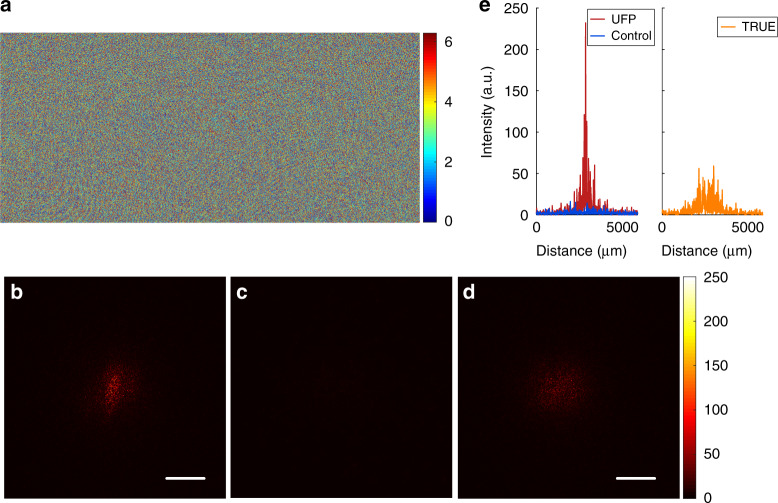
UFP optical focusing into scattering media via a full-phase modulation scheme. **a** Phase map displayed on the SLM, based on the differential signal between the measured complex fields when the ultrasound was OFF and ON. **b** Time-reversed focus from UFP optical focusing. **c** No optical focus was observed in a control experiment conducted without launching the ultrasound. **d** Time-reversed focus from conventional TRUE optical focusing under the same condition. **e** Line profiles of the central rows in **b**–**d**. Scale bars: 1 mm

We first validated UFP optical focusing into scattering media via a full-phase modulation scheme, as is widely done in conventional TRUE optical focusing. The complex fields when the ultrasound was OFF and ON were measured using four-step phase-shifting holography (see “Materials and methods”). Then we extracted the phase of the differential field between the two measured complex fields and loaded the conjugated phase on the SLM (Fig. 4a). In the playback step, the optical focus on the observation camera was observed successfully (Fig. 4b), which verified the effectiveness of the UFP optical focusing. The size of the time-reversed optical focus was approximately equal to the size of the ultrasonic focus (~1 mm for the ultrasound transducer we used). In a control experiment where we repeated the experiment above without launching the ultrasound pulse, no optical focus could be seen on the observation camera, as shown in Fig. 4c. Since both the proposed UFP optical focusing and conventional TRUE optical focusing use an ultrasonic guidestar, it is interesting to compare their focusing results. We performed TRUE optical focusing using the same experimental setup and obtained the light focus shown in Fig. 4d. Obviously, the time-reversed focus from UFP optical focusing is much brighter than that from TRUE optical focusing. The peak-to-background ratios (PBRs) of the foci in the UFP and TRUE methods are estimated to be 98 and 66, respectively. Note that we performed 3 × 3 neighborhood averaging on a focus image before calculating its peak intensity, instead of directly looking for the single pixel that had the maximum intensity. For better comparison, typical line profiles of the central rows of the foci from the two methods are presented in Fig. 4e.

The full-phase modulation scheme for UFP optical focusing makes two measurements of the complex fields, one when the ultrasound is OFF and another when it is ON. Each measurement requires four frames of phase-shifted holograms. Since many scattering media are dynamic (e.g., biological tissues), optical time reversal must be completed within the correlation time of the received optical speckles. Limited by the intrinsic speeds of such digital devices as cameras and SLMs, researchers seek to accelerate optical focusing through and into scattering media by minimizing the required data. Thanks to the differential detection scheme, UFP optical focusing can be easily realized through double-shot hologram acquisition, or even through single-shot hologram acquisition, as in the experiment described below.

**Fig. 5:**
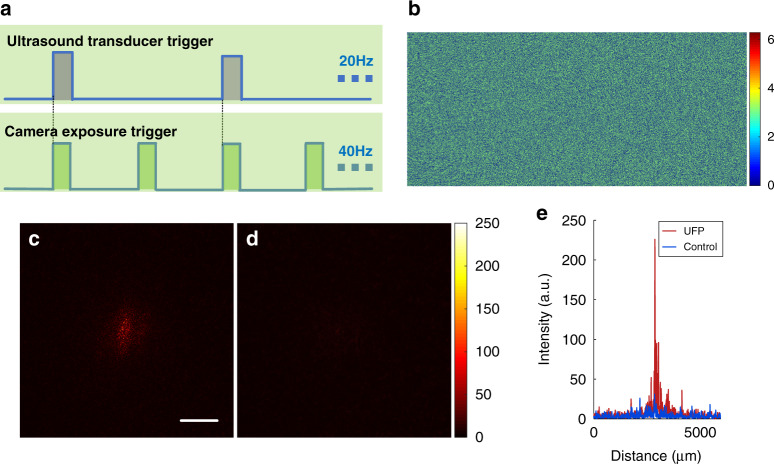
Double-shot UFP optical focusing into scattering media. **a** Time sequence for double-shot UFP optical focusing. **b** Phase map generated from the two recorded hologram frames. **c** Time-reversed focus from double-shot UFP optical focusing. **d** In a control experiment, no focus was observed on the observation camera in a double-shot measurement without ultrasound. **e** Line profiles of the central rows in **c** and **d**. Scale bar: 1 mm

The time sequence of double-shot UFP optical focusing is shown in Fig. 5a. We employed a function generator to generate two trigger signals, with repetition frequencies of 20 and 40 Hz, respectively. The low-frequency trigger signal triggered the ultrasound transducer, and the high-frequency signal triggered the camera that captured holograms. The ultrasound pulse length and the camera exposure time were both set to 1 ms. In each experiment, we captured two consecutive frames of holograms. The harmonic relationship between the two trigger signals ensured that the two consecutive holograms must be respectively captured in the ON and OFF periods of the ultrasound transducer. A differential hologram was generated via simple image subtraction of the two holograms. Finally, we obtained a binary phase map based on the differential hologram (Fig. 5b, also see “Materials and methods”) and loaded it on the SLM for wavefront modulation in the playback step. Figure 5c shows the time-reversed focus achieved by this double-shot scheme, in which a clear optical focus with a PBR of ~47 is observed. In a control experiment, we repeated the double-shot measurement without launching the ultrasound, and no focus was observed on the observation camera (Fig. 5d). The line profiles of the central rows in Fig. 5c, d are presented in Fig. 5e for better comparison.

**Fig. 6:**
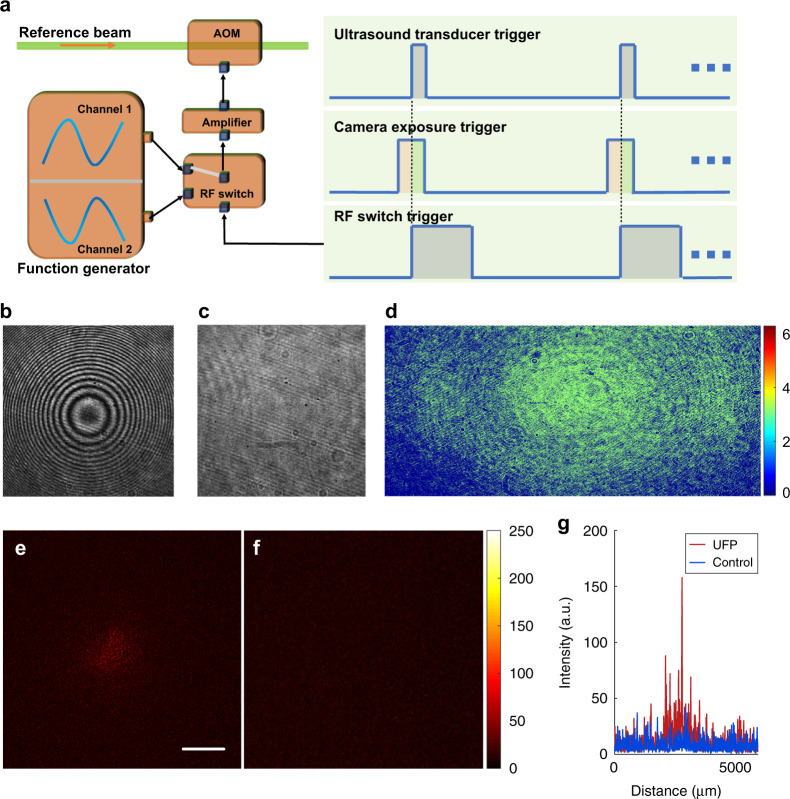
Single-shot UFP optical focusing into scattering media. **a** Time sequence for the single-shot realization of UFP optical focusing. **b** In general interferometers, if a light beam with a spherical wavefront interferes with a plane reference beam, a typical interferogram with many circular fringes will be seen. **c** In our system, because the phases of the reference beam were reversed during the first and the second halves of the camera exposure period, the interference terms canceled out completely, resulting in no interference fringes. **d** Phase map generated from the recorded single hologram. **e** Time-reversed focus from single-shot UFP optical focusing. **f** A control experiment without launching the ultrasound, yielding no focus. **g** Line profiles of the central rows in **e** and **f**. Scale bar: 1 mm

UFP optical focusing can even be implemented from only one hologram. The basic idea of single-shot UFP is to obtain the differential hologram directly during the single-exposure period of the camera, which can be realized through the time sequence presented in Fig. 6a. We employed a function generator to produce two phase-reversed sine waves that were directed to an RF switch (ZASWA-2-50DR+, Mini-Circuits). Depending on its trigger level, the RF switch selectively transmits one of the sine waves. The transmitted sine wave was amplified and sent to the AOM in the path of the reference beam. During the first half of the single-exposure period of the camera, the ultrasound transducer was OFF. At the midpoint of the single-exposure period, the ultrasound transducer was triggered to launch an ultrasound pulse, and the RF switch was also triggered to turn over its state at the same time, which resulted in phase reversal of the reference beam. Therefore, the interference terms of the integrated holograms during the first half and the second half of the exposure period had reversed signs. In this way, the whole single-exposure hologram was equivalent to the differential hologram obtained in the double-shot scheme, except for a stronger DC background in the single-shot hologram. To verify the designed configuration, we first removed the scattering medium and produced a light beam with a spherical wavefront to interfere with the reference beam. Typically, we would see an interferogram with many circular fringes on the recording camera, as presented in Fig. 6b. However, in our system, because the phases of the reference beam were reversed during the first and the second halves of the camera exposure period, the interference terms canceled out completely, resulting in no interference fringes, as demonstrated in Fig. 6c. This verification test ensured the correctness of the time sequence design in Fig. 6a. Next, we conducted normal single-shot UFP optical focusing experiments into scattering media. Similar to the data processing in the double-shot scheme, the recorded single-exposure hologram was binarized to generate a phase map displayed on the SLM for playback (Fig. 6d). Figure 6e shows a time-reversed focus from single-shot UFP optical focusing, which has a PBR of ~12. We also conducted a control experiment without launching the ultrasound, and the optical focus disappeared (Fig. 6f). The line profiles of the central rows in Fig. 6e and f are presented in Fig. 6g for better comparison.

## Discussion

We have introduced a new optical modality that uses an ultrasonic guidestar for focusing light into scattering media. In this modality, the ultrasonic guidestar also functions as a virtual dynamic guidestar through detecting ultrasound-induced optical field perturbation implied in frequency-unshifted photons. Therefore, UFP optical focusing synergistically combines the two guidestar mechanisms and offers the advantages of each simultaneously. Compared with current optical focusing methods based on physical dynamic guidestars, UFP optical focusing is noninvasive and its focus position is highly controllable. Compared with the current ultrasonic guidestar based optical focusing (i.e., TRUE optical focusing), UFP optical focusing provides better focusing performance and greater flexibility. The relative superiority of UFP optical focusing in performance has two main sources: (1) using intense zeroth-order diffracted photons to recognize the acousto-optic interaction volume, and (2) avoiding the need to extract a weak signal buried in a large background encountered in TRUE optical focusing. UFP optical focusing is also more flexible in implementation. Taking advantage of the differential detection scheme and binary phase modulation, it can be easily developed into double-shot realization and even single-shot realization for facilitating optical time reversal. In contrast, because of the strong background in holograms resulted from the untagged photons, it is much more difficult for TRUE optical focusing to work well with reduced data acquisition. We hope that UFP provides a more efficient and flexible mechanism to implement ultrasound-guided wavefront shaping for noninvasive and addressable optical focusing into scattering media.

As an ultrasound-assisted method, UFP optical focusing may work not so well in stiff media such as bones because such kinds of media would attenuate and distort the ultrasonic field severely and the ultrasonic perturbation to the media would be weaker. Of course, conventional TRUE optical focusing faces the same challenge. Therefore, we expect that the UFP method would perform better than the TRUE method in soft materials. That is because, in principle, any change induced by an ultrasonic field can cause optical field perturbation, while only a small part of the change can cause a frequency shift of photons. In the comparative experiment in Fig. 4, the sample used was a gelatin cube with a concentration of 10%. Such a gelatin concentration is commonly employed in the preparation of tissue-mimicking phantoms. We also performed tested gelatin cubes with concentrations of 20 and 30%, which were much stiffer than that in Fig. 4. These experimental results are presented in Supplementary Fig. S3. We find that the UFP method provides better focusing than the TRUE method consistently. We envision that it is also possible to extend the UFP method in other ways that can noninvasively induce change in stiff materials such as bones. For example, microwaves can penetrate deeply into stiff materials and provide local heating.

In our experiments, the practical runtimes of the full-phase modulation mode, double-shot mode and single-shot mode are about 700, 200, and 150 ms, respectively, which are determined by the speeds of the hardware, the time of data acquisition and processing, and the software control delay. For this first report on UFP wavefront shaping, we mainly focused on demonstrating the new concept and method instead of maximizing the system speed. In the demonstrated three working modes of UFP optical focusing, the full-phase modulation mode needs to measure the complex speckle field twice, so its data acquisition time is the longest. However, because complete information describing the ultrasound-induced field perturbation is measured in this mode, it has the best focusing performance. The single-shot mode has the shortest data acquisition time, at the cost of focus contrast. The double-shot mode achieves a good compromise between data acquisition time and focus contrast. These modes offer flexible choices to suit the dynamic characteristics of samples in applications. In our implementations of double-shot and single-shot UFP optical focusing, we employed only a full-grayscale phase-only SLM to perform binary phase modulation for the purpose of concept demonstration. In fact, SLMs that are specially engineered for binary modulation, such as digital micromirror devices and ferroelectric liquid crystal-based SLMs, may provide much faster response. Finally, since the area of the optical field perturbation is mainly determined by the size of the ultrasonic guidestar, an ultrasound transducer with a much tighter focus could further improve the contrast of the time-reversed optical focus.

## Materials and methods

### Details of the experimental setup

As shown in Fig. 3, the laser output (Verdi G5, Coherent) was split into two beams by a polarizing beam splitter (PBS1). The reflected beam was expanded to become a plane reference wave, while the transmitted beam illuminated the sample. A half-wave plate (HWP1) was used to control the power ratio between the reflected and transmitted beams. Two acousto-optic modulators (AOMs, AOM-505AF1, IntraAction) were mounted in the optical paths for different experimental schemes. We sandwiched a gelatin cube, which was made of porcine skin gelatin (G2500-1kG, Sigma-Aldrich, USA) and de-ionized water with a gelatin concentration of 10% by weight, between two scattering media SM1 and SM2 (Optical diffusers, DG-120, Thorlabs) to act as the sample. The gelatin cube was placed in a water tank to provide sufficient ultrasound coupling. A lab-made ultrasound transducer (3.3 MHz central frequency, 22 mm diameter, and ~1 mm focal size), driven by a power amplifier (LZY-22+, Mini-Circuits), was immersed in the water tank for transmitting a focused ultrasound field to the scattered light in the gelatin cube. For validating that light was indeed focused into the sample, a beam splitter (BS1) was inserted between SM1 and SM2 to divert a copy of the playback beam to an observation camera (Camera 2, Grasshopper 3, FLIR). The distance between SM1 and SM2 was ~10 cm to fully accommodate the water tank and BS1. Such a large gap between the two scattering media caused very severe light scattering (Supplementary Fig. S2) with negligible transmitted ballistic photons (the ratio of the power of the transmitted ballistic light to the power of the incident light was measured to be $\left( {5.1 \pm 0.2} \right) \times 10^{ - 7}$) and low light-collection efficiency. Even so, UFP optical focusing still worked well. The scattered light passing out of the sample was collected by a two-inch lens (L3) and recombined with the reference beam at BS2. Both the scattered and reference beams were reflected by an SLM (Pluto-2-VIS, Holoeye) and relayed to Camera 1 (PCO.edge 5.5, PCO) through BS3 and a camera lens (L4) for hologram recording. Note that the SLM and Camera 1 were placed in conjugated planes with a calibrated pixel-matched relationship. In the recording step, holograms were recorded by Camera 1 according to the specific data acquisition modes illustrated in “Results”. In the playback step, the sample beam was blocked by a shutter and the calculated phase pattern was loaded on the SLM to modulate the reference beam. The time-reversed optical intensity was detected on Camera 2 to validate the effectiveness of UFP optical focusing.

### Data processing

In full-phase modulation UFP optical focusing, the complex speckle fields in the OFF and ON states of the ultrasound transducer were measured by four-step phase-shifting holography. The phase shifts between holograms were introduced by a heterodyne method. Specifically, we set the driving frequency of AOM1 to be 50 MHz, and that of AOM2 to be 50.00001 MHz. In this way, the interference pattern between the scattered and reference beams was a beat signal with a 10 Hz oscillation frequency. Camera 1 recorded the beat signal at a sampling frequency of 40 Hz, so two consecutive hologram frames had a phase shift of *π*/2 radian. The measured complex optical field was reconstructed through ${{{\boldsymbol{E}}}}_m = \left( {I_1 - I_3} \right) + i(I_4 - I_2)$, where $I_1$, $I_2$, $I_3$, and $I_4$ are four *π*/2 phase-shifted holograms^46^. Finally, the phase of the differential field, ${{{\boldsymbol{E}}}}_{m\_ON} - {{{\boldsymbol{E}}}}_{m\_OFF}$, was extracted and loaded on the SLM, where ${{{\boldsymbol{E}}}}_{m\_ON}$ and ${{{\boldsymbol{E}}}}_{m\_OFF}$ are the measured complex speckle fields when the ultrasound transducer is ON and OFF, respectively.

In double-shot UFP optical focusing, the phase map on the SLM was directly calculated by the following binarization process: $$\varphi = \left\{ {\begin{array}{*{20}{c}} {0,} & {{\mathrm{if}}\;I_{{\mathrm{ON}}} - I_{{\mathrm{OFF}}} > {\mathrm{mean}}\;(I_{{\mathrm{ON}}} - I_{{\mathrm{OFF}}})} \\ {\pi ,} & {{\mathrm{if}}\;I_{{\mathrm{ON}}} - I_{{\mathrm{OFF}}} \le {\mathrm{mean}}\;(I_{{\mathrm{ON}}} - I_{{\mathrm{OFF}}})} \end{array}} \right.$$, where $I_{{\mathrm{ON}}}$ and $I_{{\mathrm{OFF}}}$ are the holograms captured when the ultrasound transducer is ON and OFF, respectively, and mean () represents the average operator. In single-shot UFP optical focusing, the phase map on the SLM was calculated through a similar binarization process: $$\varphi = \left\{ {\begin{array}{*{20}{c}} {0,} & {{\mathrm{if}}\;I > {\mathrm{mean}}\;(I)} \\ {\pi ,} & {{\mathrm{if}}\;I \le {\mathrm{mean}}\;(I)} \end{array}} \right.$$, where *I* is the captured single-shot hologram. See supplementary notes for more illustrations.

### Simulation details

In the simulations, the scattering medium was composed of 401 thin scattering layers uniformly spaced with a tiny distance, Δ*l*, between them (Δ*l* = 20 μm in our case). We assumed that light was scattered only by these scattering layers, while it experienced non-scattering free space propagation between neighboring layers. Each scattering layer was divided into 1001 × 1001 pixel grids with a 5 μm pixel size. A randomly distributed refractive index map, $n\left( {x,y} \right)$, was allocated to each scattering layer, with a mean $n_0 = 1.4$ and standard deviation $\sigma (n_0) = 0.003$. The incident light, which had a wavelength of 532 nm and a beam diameter of 2 mm, illuminated the first scattering layer of the scattering medium. In the central volume of the scattering medium, an ultrasound field propagating perpendicularly to the optical axis was simulated. The focal diameter of the ultrasound field was 80 μm, and the length of focal area along the acoustic axis was 300 μm. The ultrasonic frequency and peak pressure were set to be 1 MHz and 2 MPa, respectively. The adiabatic piezo-optical coefficient of the medium was $\partial n/\partial p = \eta /\rho v_a^2$, where $\eta = 0.32$ is the elasto-optical coefficient of water at room temperature, $\rho = 10^3\,{\mathrm{kg}} \cdot {\mathrm{m}}^{ - 3}$ is the mass density of the medium, and $v_a = 1480\,{\mathrm{m}} \cdot {\mathrm{s}}^{ - 1}$ is the ultrasound velocity. A collecting lens with a focal length of 40 mm was placed behind the scattering medium to collect the scattered light. The distance between the collecting lens and the scattering medium was set to be 30 mm. Finally, an observation plane was located 80 mm away from the collecting lens, where the optical field of the scattered light was recorded and analyzed.

When light passed through one of the thin scattering layers, a random phase factor $P\left( {x,y} \right) = 2\pi n\left( {x,y} \right){{{\mathrm{{\Delta}}} }}l/\lambda$ was added to the optical field, where *λ* is the wavelength of the light. Then it propagated a distance Δ*l* to the next scattering layer. The collecting lens was modeled by a phase modulation function, ${{{\mathrm{exp}}}}[ - i\pi (x^2 + y^2)/\lambda f]$, where *f* is the focal length of the lens. Keeping these different phase modulations in mind, we can use an angular spectrum propagation method to calculate the complex optical fields in different planes^47,48^. Specifically, optical propagation in free space is equivalent to an optical frequency modulation with a frequency transfer function, $$H\left( {f_x,f_y} \right) = \left\{ {\begin{array}{*{20}{r}} \hfill {{\mathrm{exp}}\left[ {ikd\sqrt {1 - \left( {\lambda f_x} \right)^2 - \left( {\lambda f_y} \right)^2} } \right],} & \hfill {f_x^2 + f_y^2 \le 1/\lambda ^2}\\ \hfill {0,} & \hfill {{\mathrm{otherwise}}}\end{array}} \right.$$, where *k* is the wavenumber of the light, $f_x$ and $f_y$ are the two-dimensional (2D) spatial frequencies, and *d* is the propagation distance. We first got the original frequency distribution of an optical field by a 2D Fourier transformation and then multiplied it by the frequency transfer function above to obtain the new frequency distribution in a target plane. Finally, 2D inverse Fourier transformation was performed to determine the optical field distribution in the space domain. In practical implementations of the angular spectrum propagation method, we employed fast Fourier transformation (FFT) to facilitate calculations. Note that, since FFT implies circular convolution rather than linear convolution in the space domain, applying zero padding to the data in the space domain is generally required to avoid edge errors from FFT^49^.

Since the ultrasound field in the scattering medium is oscillating with time, we first calculated the optical fields in the observation plane at different sampling times, $t_0$, $t_1$,…$t_n$. The time sequence of optical fields at each pixel of the observation plane was then Fourier transformed to obtain the amplitude and phase of the different frequency components of photons. The total sampling time determines the frequency resolution, while the sampling time interval determines the maximum frequency range that can be extracted. In our simulations, we set the total sampling time as 10 μs, with a 0.1 μs interval.

## Supplementary information

Supplementary information
