# Supplementary material for: Focusing light into scattering media with ultrasound-induced field perturbation
Source: Light Sci Appl. 2021 Aug 2;10:159. doi: 10.1038/s41377-021-00605-7 (PMC8329210; doi:10.1038/s41377-021-00605-7)
Supplement: Supplementary file 1 — Supplementary information [file 41377_2021_605_MOESM1_ESM.pdf]

Supplementary Information for

# Focusing light into scattering media with ultrasound-induced field perturbation

**Zhongtao Cheng and Lihong V. Wang***

*Caltech Optical Imaging Laboratory, Andrew and Peggy Cherng Department of Medical Engineering, Department of Electrical Engineering, California Institute of Technology, Pasadena, California 91125, USA*

**Corresponding author: LVW@caltech.edu*

**Supplementary Note 1: Mathematical illustration of double-shot UFP optical focusing**

In double-shot UFP optical focusing, we cannot measure the complex optical field when the ultrasound is ON or OFF from only two holograms. Instead, we employ the differential intensity hologram to generate a binary phase map for optical time reversal. The two holograms recorded can be respectively expressed as

$$I_{ON} = (\boldsymbol{E}_{m\_ON} + \boldsymbol{E}_R)(\boldsymbol{E}_{m\_ON} + \boldsymbol{E}_R)^* = \left|\boldsymbol{E}_{m\_ON}\right|^2 + |\boldsymbol{E}_R|^2 + \boldsymbol{E}_{m\_ON}{}^*\boldsymbol{E}_R + \boldsymbol{E}_{m\_ON}\boldsymbol{E}_R{}^*, \quad \text{(S1)}$$

and

$$I_{OFF} = (\boldsymbol{E}_{m\_OFF} + \boldsymbol{E}_R)(\boldsymbol{E}_{m\_OFF} + \boldsymbol{E}_R)^* = \left|\boldsymbol{E}_{m\_OFF}\right|^2 + |\boldsymbol{E}_R|^2 + \boldsymbol{E}_{m\_OFF}{}^*\boldsymbol{E}_R + \boldsymbol{E}_{m\_OFF}\boldsymbol{E}_R{}^*. \quad \text{(S2)}$$

Here, $\boldsymbol{E}_{m\_ON}$ and $\boldsymbol{E}_{m\_OFF}$ are the optical fields in the measurement plane when the ultrasound is ON and OFF, respectively; $\boldsymbol{E}_R$ is the reference field. Thus, the differential intensity hologram is

$$\begin{aligned}\Delta I &= \left|\boldsymbol{E}_{m\_ON}\right|^2 - \left|\boldsymbol{E}_{m\_OFF}\right|^2 + (\Delta\boldsymbol{E})^*\boldsymbol{E}_R + (\Delta\boldsymbol{E})\boldsymbol{E}_R{}^* \\ &= \left|\boldsymbol{E}_{m\_ON}\right|^2 - \left|\boldsymbol{E}_{m\_OFF}\right|^2 + 2|\Delta\boldsymbol{E}||\boldsymbol{E}_R|\cos(\varphi_\Delta),\end{aligned} \quad \text{(S3)}$$

where $\Delta\boldsymbol{E} = \boldsymbol{E}_{m\_ON} - \boldsymbol{E}_{m\_OFF}$; $|\Delta\boldsymbol{E}|$ and $\varphi_\Delta$ are the amplitude and phase of $\Delta\boldsymbol{E}$, respectively. Note that we have assumed the phase of the reference field to be zero.

As given in the manuscript, $\boldsymbol{E}_{m\_ON} = \mathbf{T}(\boldsymbol{E}_{t\_o} + \boldsymbol{E}_{t\_p})$ and $\boldsymbol{E}_{m\_OFF} = \mathbf{T}\boldsymbol{E}_{t\_o}$, where $\boldsymbol{E}_{t\_o}$ is the original optical field when the ultrasonic field is absent and $\boldsymbol{E}_{t\_p}$ is the field perturbation induced by the ultrasound; $\mathbf{T}$ is the transmission matrix describing the scattering medium. Accordingly, we have

$$\left|\boldsymbol{E}_{m\_ON}\right|^2 - \left|\boldsymbol{E}_{m\_OFF}\right|^2 = \left|\mathbf{T}\boldsymbol{E}_{t\_p}\right|^2 + 2\left|\mathbf{T}\boldsymbol{E}_{t\_o}\right|\left|\mathbf{T}\boldsymbol{E}_{t\_p}\right|\cos(\varphi_{o\_p}), \quad \text{(S4)}$$

where $\varphi_{o\_p}$ is the phase difference between fields $\mathbf{T}\boldsymbol{E}_{t\_o}$ and $\mathbf{T}\boldsymbol{E}_{t\_p}$. In double-shot UFP optical focusing, we need to calculate $\Delta I - mean(\Delta I)$ first, and then binarize the result to 0 or $\pi$ using

zero as the threshold. Because the phase terms $\varphi_\Delta$ and $\varphi_{o\_p}$ are both uniformly distributed among $[0,2\pi)$, it can be derived that

$$mean(\Delta I) = mean\left(\left|\mathbf{T}\boldsymbol{E}_{t\_p}\right|^2\right), \tag{S5}$$

and

$$\Delta I - mean(\Delta I) = \left|\mathbf{T}\boldsymbol{E}_{t\_p}\right|^2 - mean\left(\left|\mathbf{T}\boldsymbol{E}_{t\_p}\right|^2\right) + 2\left|\mathbf{T}\boldsymbol{E}_{t\_o}\right|\left|\mathbf{T}\boldsymbol{E}_{t\_p}\right|\cos(\varphi_{o\_p})$$
$$+2\left|\mathbf{T}\boldsymbol{E}_{t\_p}\right||\boldsymbol{E}_R|\cos(\varphi_\Delta). \tag{S6}$$

Since the ultrasound-induced field perturbation $\boldsymbol{E}_{t\_p}$ is only non-zero within the compact ultrasonic focus, without loss of generality, we assume that the first element of $\boldsymbol{E}_{t\_p}$ represents an optical mode within the ultrasonic focus, while all the other elements are zeros. That is, we consider a simplified $\boldsymbol{E}_{t\_p}$ as

$$\boldsymbol{E}_{t\_p} = (A_p\ 0\ 0\ \cdots 0)^T_{1\times J}, \tag{S7}$$

where "$T$" represents vector transpose and $J$ is the total number of vector elements. For the convenience of the following derivation, we also express $\boldsymbol{E}_{t\_o}$ explicitly as

$$\boldsymbol{E}_{t\_o} = (e_1\ e_2\ e_3\ \cdots e_J)^T_{1\times J}. \tag{S8}$$

Based on Eqs. (S7) and (S8), we can write the $m$-th elements of the fields $\mathbf{T}\boldsymbol{E}_{t\_p}$ and $\mathbf{T}\boldsymbol{E}_{t\_o}$ as

$$(\mathbf{T}\boldsymbol{E}_{t\_p})^{(m)} = A_p t_{m1}, \tag{S9}$$

$$(\mathbf{T}\boldsymbol{E}_{t\_o})^{(m)} = \sum_{k=1}^{J} t_{mk}e_k, \tag{S10}$$

where $t_{mj}$ is the element of matrix $\mathbf{T}$ (i.e., $\mathbf{T} = (t_{mj})_{M\times J}$ ), which follows a circular Gaussian distribution. Therefore, the $m$-th element of $\Delta I - mean(\Delta I)$ is

$$[\Delta I - mean(\Delta I)]^{(m)} = {A_p}^2[|t_{m1}|^2 - mean(|t_{m1}|^2)] + 2A_p|t_{m1}|\left|\sum_{k=1}^{J} t_{mk}e_k\right|\cos\left({\varphi_{o\_p}}^{(m)}\right)$$
$$+2|\boldsymbol{E}_R|A_p|t_{m1}|\cos(\varphi_{m1})$$

$$\approx 2|\boldsymbol{E}_R||A_p||t_{m1}|\cos(\varphi_{m1}), \tag{S11}$$

where $\varphi_{m1}$ is the phase of the complex element $t_{m1}$. Because $|t_{mj}|$ is much less than unity, the terms in the first line of Eq. (S11) is negligible if the cosine term in the second line is not too close to zero, the violation of which occurs only with a low probability for a fully developed speckle field. When $[\Delta I - mean(\Delta I)]^{(m)} > 0$, we can infer that $\varphi_{m1}$ is between $[-\pi/2, \pi/2]$. Otherwise, $\varphi_{m1}$ is between $[\pi/2, 3\pi/2]$. We can see that the quantity $\Delta I - mean(\Delta I)$ approximately indicates the information about the range of $\varphi_{m1}$.

In the playback step, the reference beam is modulated by an SLM with a phase pattern $\varphi_{SLM}$. The playback optical field at the target plane can be expressed as

$$\boldsymbol{E}_P = \mathbf{T}^T|\boldsymbol{E}_R|\exp(i\varphi_{SLM}). \tag{S12}$$

The $j$-th element of $\boldsymbol{E}_P$ is

$$\boldsymbol{E}_P{}^{(j)} = \sum_{m=1}^{M}|t_{mj}||\boldsymbol{E}_R|\exp\left[i(\varphi_{mj} + \varphi_{SLM}{}^{(m)})\right]. \tag{S13}$$

Here, $\varphi_{SLM}{}^{(m)}$ is the $m$-th element of $\varphi_{SLM}$. According to the binary processing introduced in the Methods section of the manuscript, we know that $\varphi_{SLM}$ is determined by

$$\varphi_{SLM}{}^{(m)} = \begin{cases} 0, if\ \varphi_{m1} \in [-\pi/2, \pi/2] \\ \pi, if\ \varphi_{m1} \in [\pi/2, 3\pi/2] \end{cases}. \tag{S14}$$

As a result, the phase terms of all the phasors in the summation of $\boldsymbol{E}_P{}^{(1)}$ are in the range of $[-\pi/2, \pi/2]$. Thus, these phasors are aligned to interfere constructively, which forms the time-reversed focus. For $j \neq 1$, the phase terms in the summation of $\boldsymbol{E}_P{}^{(j)}$ are still uniformly distributed between $[0, 2\pi)$, which results in background speckles.

**Supplementary Note 2: Mathematical illustration of single-shot UFP optical focusing**

The single-shot UFP optical focusing generates a differential intensity hologram directly during the single-exposure period of the camera. Because the phase of the reference beam is reversed during the second half of the exposure period of the camera, we can express the recorded single-shot hologram as

$$I = (\boldsymbol{E}_{m\_ON} + \boldsymbol{E}_R)(\boldsymbol{E}_{m\_ON} + \boldsymbol{E}_R)^* + (\boldsymbol{E}_{m\_OFF} - \boldsymbol{E}_R)(\boldsymbol{E}_{m\_OFF} - \boldsymbol{E}_R)^*$$

$$= 2|\boldsymbol{E}_R|^2 + \left(\left|\boldsymbol{E}_{m\_ON}\right|^2 + \left|\boldsymbol{E}_{m\_OFF}\right|^2\right) + (\Delta\boldsymbol{E})^*\boldsymbol{E}_R + (\Delta\boldsymbol{E})\boldsymbol{E}_R{}^*$$

$$= 2|\boldsymbol{E}_R|^2 + \left(\left|\boldsymbol{E}_{m\_ON}\right|^2 + \left|\boldsymbol{E}_{m\_OFF}\right|^2\right) + 2|\Delta\boldsymbol{E}||\boldsymbol{E}_R|\cos(\varphi_\Delta). \tag{S15}$$

Here, we can see that the whole single-exposure hologram was equivalent to the differential hologram obtained in the double-shot scheme [Eq. (S3)], except for a stronger DC background in the single-shot hologram. After subtracting the mean of the hologram, we obtain

$$I - mean(I) = \left(\left|\boldsymbol{E}_{m\_ON}\right|^2 + \left|\boldsymbol{E}_{m\_OFF}\right|^2\right) - mean\left(\left|\boldsymbol{E}_{m\_ON}\right|^2 + \left|\boldsymbol{E}_{m\_OFF}\right|^2\right)$$

$$+2\left|\mathbf{T}\boldsymbol{E}_{t\_p}\right||\boldsymbol{E}_R|\cos(\varphi_\Delta)$$

$$= \left|\mathbf{T}\boldsymbol{E}_{t\_p}\right|^2 - mean\left(\left|\mathbf{T}\boldsymbol{E}_{t\_p}\right|^2\right) + 2\left[\left|\mathbf{T}\boldsymbol{E}_{t\_o}\right|^2 - mean\left(\left|\mathbf{T}\boldsymbol{E}_{t\_o}\right|^2\right)\right]$$

$$+2\left|\mathbf{T}\boldsymbol{E}_{t\_o}\right|\left|\mathbf{T}\boldsymbol{E}_{t\_p}\right|\cos\left(\varphi_{o\_p}\right)$$

$$+2\left|\mathbf{T}\boldsymbol{E}_{t\_p}\right||\boldsymbol{E}_R|\cos\left(\varphi_\Delta\right). \tag{S16}$$

Equation (S16) is also similar to Eq. (S6), so we can follow the subsequent derivations as in the double-shot case, which will not be repeated here. Finally, we will obtain

$$[I - mean(I)]^{(m)} \approx 2|\boldsymbol{E}_R|A_p|t_{m1}|\cos(\varphi_{m1}). \tag{S17}$$

Therefore, we can still approximately infer the range of $\varphi_{m1}$ from the single-shot hologram, as done in Eq. (S11). Comparing Eq. (S16) with Eq. (S6) shows that the single-shot hologram has an extra term $2\left[\left|\mathbf{T}\boldsymbol{E}_{t\_o}\right|^2 - mean\left(\left|\mathbf{T}\boldsymbol{E}_{t\_o}\right|^2\right)\right]$. Although this extra term is also much smaller

than the term $2\left|\mathbf{T}\boldsymbol{E}_{t\_p}\right|\left|\boldsymbol{E}_R\right|\cos(\varphi_\Delta)$, it makes the approximation of Eq. (S17) more inaccurate than that of Eq. (S11), which explains why the double-shot UFP optical focusing has better performance than the single-shot UFP optical focusing.

## Supplementary Figures

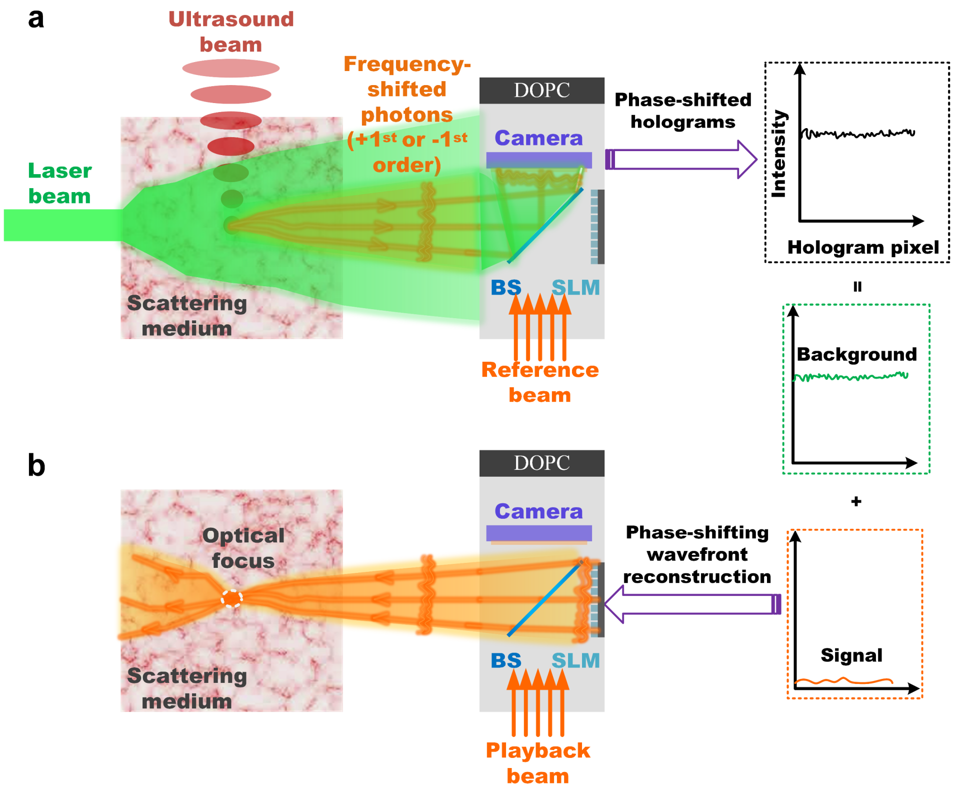


**Figure S1. Schematic illustration of the conventional TRUE optical focusing. a**, In conventional TRUE optical focusing, only the first-order scattered photons (denoted by the orange color) frequency-shifted by acousto-optic modulation are considered useful. Their optical field is recorded through interference with an equally frequency-shifted reference beam. Since the first-order photons occupy only a small proportion of the whole optical field reaching the camera plane, the recorded hologram is composed of a strong speckle background created by powerful non-first-order photons and a weak useful signal from the first-order frequency-shifted photons. Retrieving such a small signal overwhelmed by a strong background generally requires more data acquisition (e.g., many frames of holograms with phase shifts) and is susceptible to noise.

**b**, The retrieved wavefront of the frequency-shifted scattered photons is phase-conjugated and played back through a DOPC system to realize optical focusing.

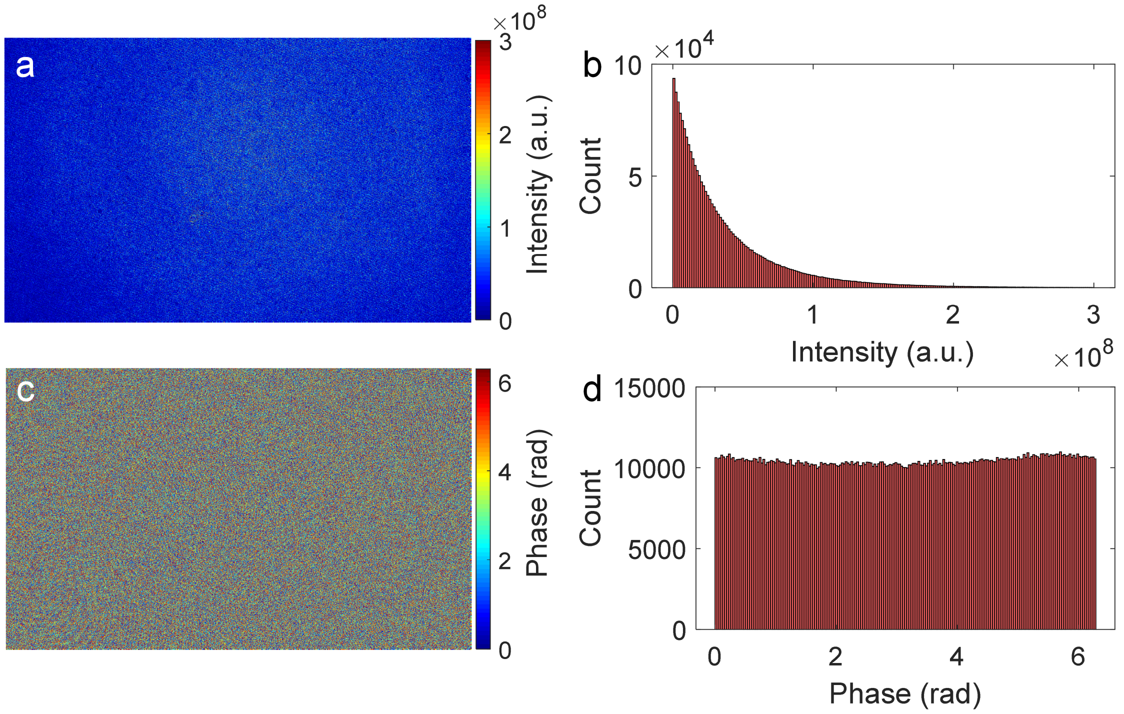


**Figure S2. A typical speckle field measured in our experiments and the statistical distributions of its intensity and phase. a-b,** The intensity of the speckle field and its statistical distribution, respectively. **c-d**, The phase of the speckle field and its statistical distribution, respectively. It can be seen that the intensity and phase of the speckle field approximately follow the negative exponential distribution and $[0, 2\pi)$ uniform distribution, respectively, which are the hallmarks of a fully developed speckle field.

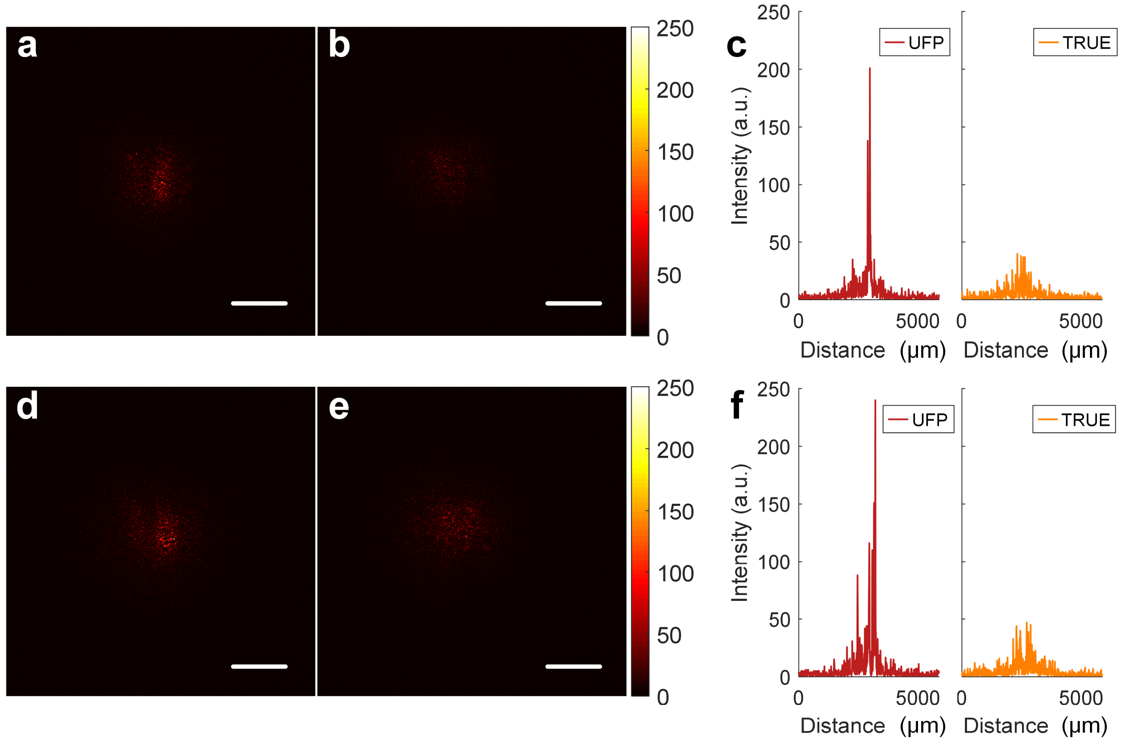


**Figure S3. Focusing into samples with different stiffnesses. a-c**, Results for a gelatin cube with a gelatin concentration of 20%. **a**, Time-reversed focus from UFP optical focusing. **b**, Time-reversed focus from conventional TRUE optical focusing under the same condition. **c**, Line profiles of the central rows in **a** and **b**. The PBRs of the foci in **a** and **b** are 95 and 55, respectively. **d-f**, Same as **a-c** correspondingly but for a gelatin cube with a gelatin concentration of 30%. The PBRs of the foci in **d** and **e** are 105 and 65, respectively. Scale bars: 1 mm. From the results here as well as that in Fig. 4 of the main manuscript, we find that the two methods do not depend on the gelatin concentration significantly, and the UFP method provides better focusing than the TRUE method consistently.
